# Supplementary material for: Quantifying the rise and fall of scientific fields
Source: PLoS One. 2022 Jun 23;17(6):e0270131. doi: 10.1371/journal.pone.0270131 (PMC9223313; doi:10.1371/journal.pone.0270131)
Supplement: S1 File — (PDF) [file pone.0270131.s001.pdf]

Supplementary Information: Quantifying the rise and fall of scientific fields

Chakresh Kumar Singh[1,2], Emma Barme[1,2], Robert Ward[3], Liubov Tupikina[1,2,4], Marc Santolini[1,2*],

1 Université Paris Cité, Inserm, System Engineering and Evolution Dynamics, F-75004 Paris, France
2 Learning Planet Institute, F-75004 Paris, France
3 School of Public Policy, Georgia Institute of Technology, Atlanta, GA 30332
4 Nokia Bell labs, France

* Corresponding author: marc.santolini@cri-paris.org

1 arXiv as a dataset

Paul Ginsparg created arXiv in 1991. It was initially designed for sharing preprint articles with friends and colleagues [1]. The reasons why researchers favor uploading their articles on arXiv are diverse. With a low threshold in the review phase and a minimal time between submission and online appearance, it provides a fast way for researchers to share their results with the scientific community. This in turn helps them in getting feedback from the larger ecosystem and gain intellectual precedence for their claims. The management team of arXiv follows a strict and systematic procedure ensuring accurate classification of an article to its subject domain (see Field tags management). Though arXiv is lenient in its quality control as compared to a stricter "peer-reviewed" system, an earlier study reports that $\sim 64\%$ arXiv articles end up publishing in WOS (Web Of Science) indexed journals and many journals also have started accepting arXiv preprint for submissions [2], supporting the credibility of arXiv articles.

2 Field tags management

Users can choose appropriate field tags for their articles from the existing ones. They, however, cannot create their tags. The tags assigned by users are then reviewed by moderators of different subject domains and changed if deemed necessary. New field tags can only be introduced by the arXiv administration. They do consider proposals from researchers for introducing new tags and only after considering multiple factors such as the size of the research community, frequency of articles appearing in the field, or its impact on arXiv. A recent example of this was the introduction of two new tags in 2018: econ.TH and econ.GN, corresponding to Economics Theory and Economics General. This happened after a community of economists proposed it to arXiv. However, most of the field tags appeared in the initial years (see Fig S2).

3 Growth rate

To calculate the growth rate of the arXiv dataset, we consider the growth function as defined in Eq1, with growth rate r:

$$N(t) = N_0 e^{rt} \tag{1}$$

We then fit the cumulative number of articles and number of authors in the dataset over time as shown in Fig S1.

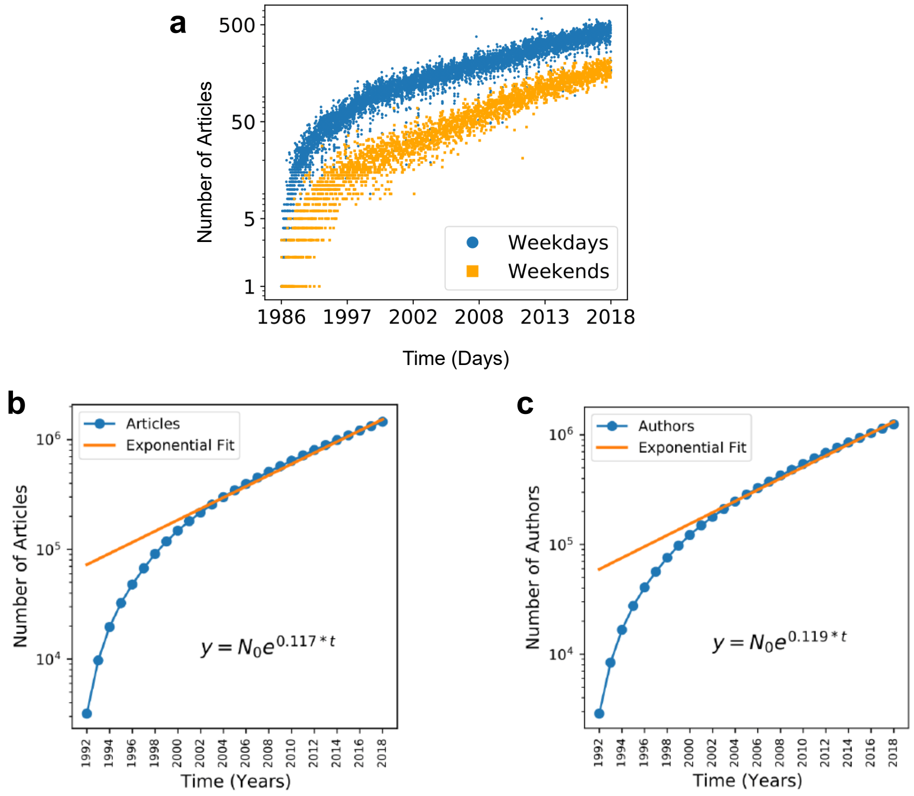

Fig SI 1. **a** Cumulative number of articles submitted to arXiv in time. **b** Cumulative number of (unique) authors. Both number of articles and of authors grow exponentially with a doubling period of $\sim$ 6 years.The daily number of articles submitted to arXiv since 1986 shows an exponential growth over time, with a doubling period of 6 years. The data also shows strong seasonality with 10 times fewer articles over the weekends.

The growth rates r for articles and authors are respectively 0.117 and 0.119. Hence the doubling period i.e $\frac{ln2}{r}$ for articles and authors is resp. 5.9 and 5.8 years.

3.1 Yearly proportion of articles for every field

The proportion of articles histogram Fig S2a observes a shift in research interest toward Computer Science fields. We also show in Fig S2b that the number of unique field tags remain constant post 1997. With no new tags the incoming papers every year are distributed within the existing tags. Evolution of fields in such a closed system can be viewed by the proportion of articles each field tag shares per year. We observe that this proportion observes a rise and fall pattern which when delineated into different stages of evolution exhibits universal trends in the evolution of scientific fields.

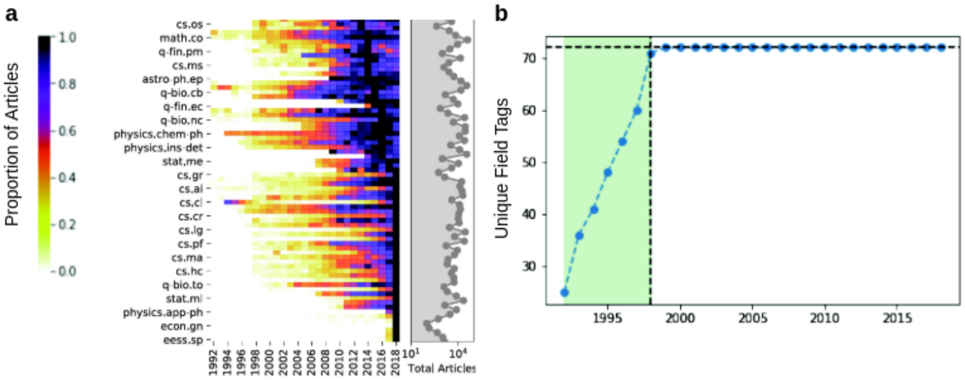

Fig SI 2. (a) Zoom of histogram in Fig 1b of the main manuscript exhibits peaking of field tags from the field of computer science in later years, including CS.AI (artificial intelligence) (b) Cumulative number of field tags across time, among the 72 studied. After an initial growth in the early years, the number of unique tags stays constant

4 Fitting the empirical data

Normalizing the Gumbel Distribution Function

Since for each field we only observe a finite sampling period of the full distribution, we need to normalize the Gumbel distribution between times t_1 and t_2 to improve the fit. Given the Gumbel function $G(x)$, we find the normalizing constant such that:

$$C \int_{t_1}^{t_2} G(x, \alpha, \beta) dx = 1 \tag{2}$$

$$C \int_{t_1}^{t_2} \frac{1}{\beta} e^{\frac{-(x-\alpha)}{\beta}} e^{-e^{\frac{-(x-\alpha)}{\beta}}} dx = 1 \tag{3}$$

Let $y = e^{-\frac{(x-\alpha)}{\beta}} \implies dy = -\frac{1}{\beta} e^{-\frac{(x-\alpha)}{\beta}} dx$. Replacing above in Eq.3 and adjusting limits we get:

$$C \int_{e^{-\frac{(t_1-\alpha)}{\beta}}}^{e^{-\frac{(t_2-\alpha)}{\beta}}} -e^{-y} dy = 1 \tag{4}$$

$$C e^{-y} \Big|_{e^{-\frac{(t_1-\alpha)}{\beta}}}^{e^{-\frac{(t_2-\alpha)}{\beta}}} = 1 \tag{5}$$

$$C \left[e^{-e^{-\frac{(t_2-\alpha)}{\beta}}} - e^{-e^{-\frac{(t_1-\alpha)}{\beta}}} \right] = 1 \tag{6}$$

$$C = \frac{1}{\left[e^{-e^{-\frac{(t_2-\alpha)}{\beta}}} - e^{-e^{-\frac{(t_1-\alpha)}{\beta}}} \right]} \tag{7}$$

With the above C value we can normalize the Gumbel distribution function for any values of t_1 and t_2. Note that when $t_1 \to -\infty$ and $t_2 \to \infty$ the constant $C \to 1$.

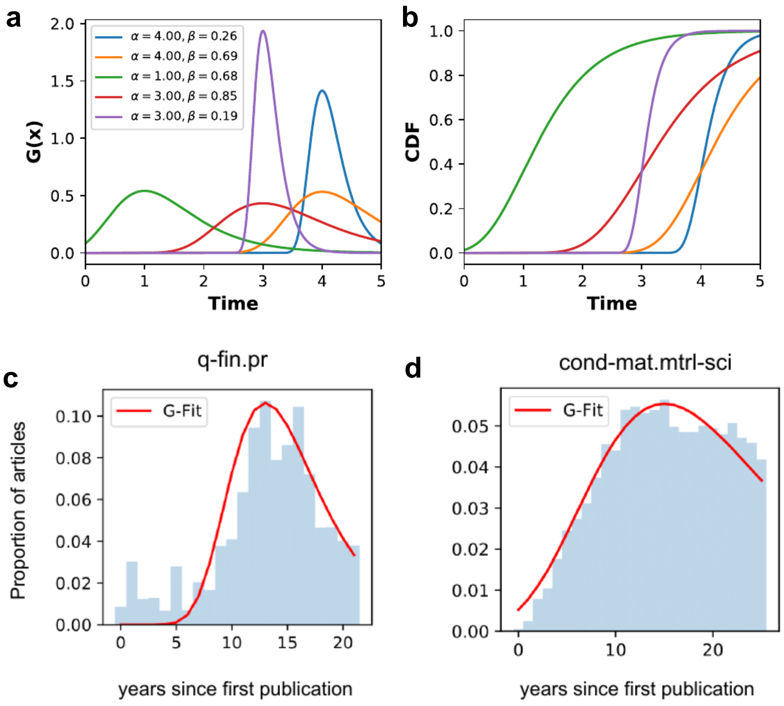

Fig SI 3. (a) and (b) show examples of Gumbel distributions for different location and shape parameter values with (a) showing the PDF and (b) showing the CDF. (c) Empirical distribution and Gumbel fits for the fields of (c) Quantitative Finance (q-fin.pr) and (d) Condensed matter material sciences (cond-mat.mtrl-sci).

References

1. Ginsparg P. ArXiv at 20. Nature. 2011;476(7359):145–147.
2. Larivière V, Sugimoto CR, Macaluso B, Milojević S, Cronin B, Thelwall M. arXiv E-prints and the journal of record: An analysis of roles and relationships. Journal of the Association for Information Science and Technology. 2014;65(6):1157–1169.

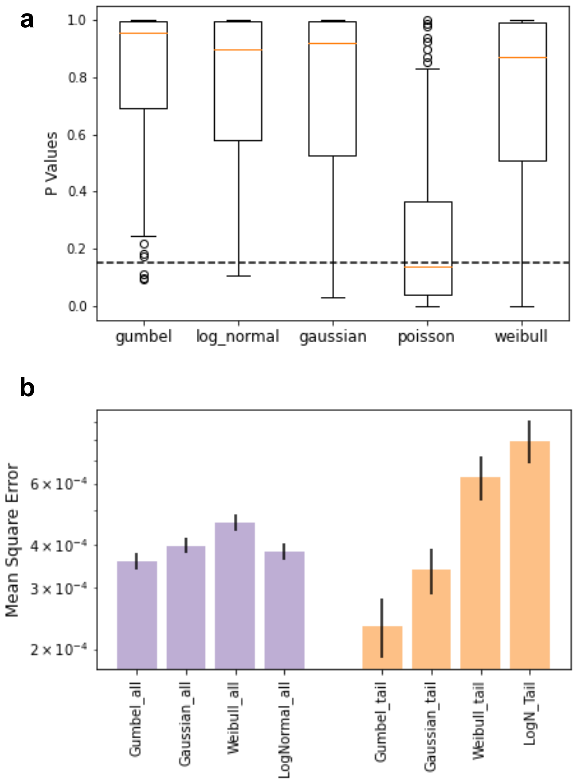

Fig SI 4. (a) We compare the p-values of Kolmogorov-Smirnov fitness statistics using different distributions functions. Higher p-values indicate better fitness. We observe that two parameter functions fit better than single parameter functions. (b) Further, we show the Mean Square Error using the true vs. predicted values for Gumbel, Gaussian, Weibull and Lognormal distributions considering all data (purple) and considering the right tail defined as the 5% of points in the highest re-scaled time range (orange). We observe that gumbel distribution captures the data better than other distributions.

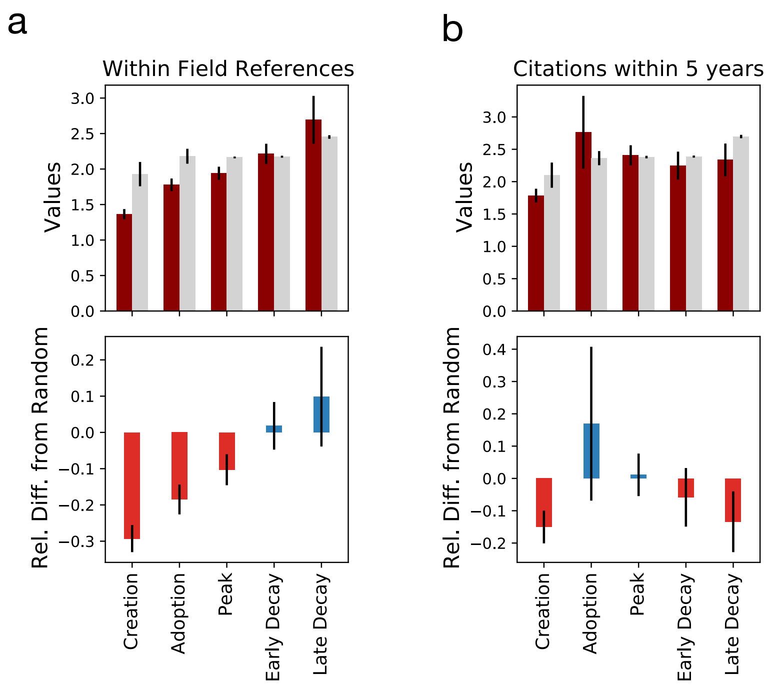

Fig SI 5. Same as Fig 3a, for references (**a**) and citations (**b**) within the same field than the article. Citations are limited to the 5 years following the article.

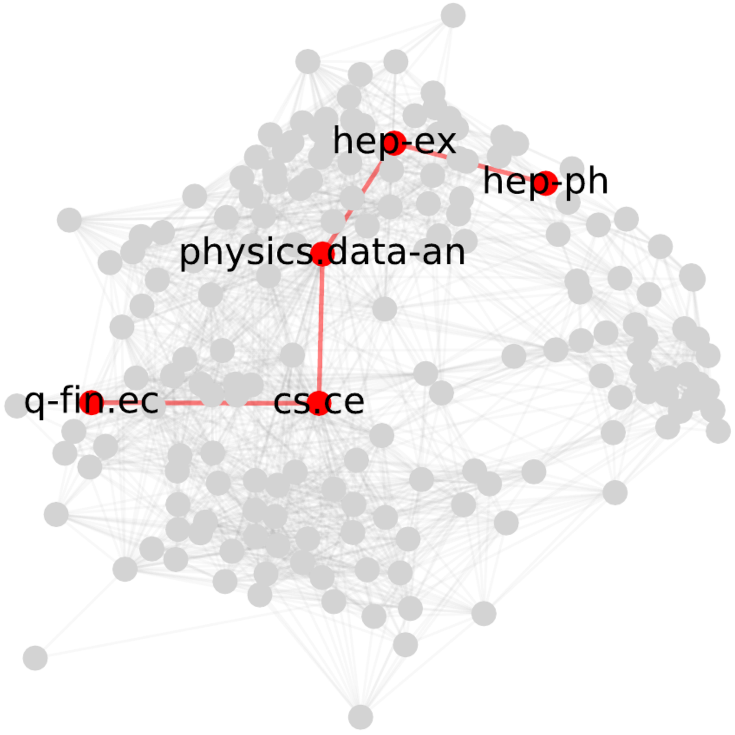

Fig SI 6. Example of a shortest path linking the distant fields of Quantitative finance and High Energy Physics in the field co-occurrence network.
