# Supplementary material for: Quantifying the rise and fall of scientific fields
Source: PLoS One. 2022 Jun 23;17(6):e0270131. doi: 10.1371/journal.pone.0270131 (PMC9223313; doi:10.1371/journal.pone.0270131)
Supplement: Attachment — Submitted filename: version1.pdf [file pone.0270131.s003.pdf]

This paper tackles the question on quantifying the dynamics of various scientific fields. The question is addressed by performing a statistical analysis and modelling of a dataset of well-annotated fields tags in Computer science, Mathematics, Finance and Biology, where for 1.45 million articles from 175 research fields in arXiv repository are used. The authors use Gumbel distribution as a proxy to model the field temporal distributions. It is a new and simple scale-invariant methods in quantifying evolution of scientific fields. Then the statistical analysis shows the general characteristics of articles and authors across evolution stages of fields: the early phase is represented by the mixing of cognitively distant fields by small teams of interdisciplinary authors, while late phases exhibit the role of specialized, large teams building on the previous works in the field.

The method and results are presented well using appropriate metrics and figures. The claims of the paper are supported by the results.

Everything looks technically correct and the language is clear.

I have two major issues and a few minor concerns regarding the analysis and interpretation of some of the results:

Major issues:

1. In order to control the growth effect over time of arXiv articles, the authors use yearly proportion of articles of considered field in arXiv as a metric to delineate the temporal characters instead of absolute number. My concern on this metric is that papers in some big fields will be like to be overwhelmed to small fields resulting in the misunderstanding of real evolution trend of small fields. For example, we can see from fig 2d, Computer science recently enters in decay stage. However, the empirical intuition is that computer science is booming specially in AI field. The phenomenon could also happen in other fields. The authors are suggested to further clarify the reasonable usage of this metric to eliminate reader’s worries.

2. By statistical analysis highlighting characteristics shared by articles and authors across various stages of a field evolution, the authors draw one of the main conclusions: early stages are characterized by small teams and interdisciplinary authors, while late stages exhibit the role of large, more specialized teams. They wrote in Discussion section of the paper "This supports the general finding that small teams disrupt while large teams develop science and technology [33, 34]." Although I agree the narration above, I also suggest that the authors make an extra effort to calculate the Disruptiveness Index to support their findings.

And there is a recently published paper closely related to the topic in this paper, it is recommended to be added in the reference list.

Johan S. G. Chu, James A. Evans. (2021). Slowed canonical progress in large fields of science. Proc Natl Acad Sci, 41(118), e2021636118.

A few minor concerns:

3.The paper uses re-scaled Gumbel distribution to model the rise and fall of a scientific filed. I have a detailed doubt about the calculation of stages. In fig 2b and fig 2d, if they use the 25%, 16%, 50% and 84% quantiles of the Gumbel distribution as cutpoints of stage, the math is recently on the decay stage. But at 84% quantiles, it is around the peak rather than late decline in fig 2b. Maybe I miss the details about the method. The authors are suggested to explain this point clearly.

4.Under strict conditions for data: longevity, unimodality and completeness, 175 research fields are reduced the number of fields to 72. The exiting rate of fields is bigger than ½, which means the authors lose and neglect lots of information in the main text. In my opinion, while it is much meaningful to find the scale-invariant patterns in the evolution of many scientific fields, the manuscript can be better if the authors clearly highlight the limitation in Discussion. The evolution characters of 72 fields in the paper cannot stand for the pattern of all fields in science. And it is worth exploring the untrivial patterns in further research.

5. In fig 1b, the authors compare the publishing seasonality between weekdays and weekends while the information is not mentioned or discussed at all to support the results in the manuscript. In my opinion, it is redundant and useless for publication.

6. The Fig 2d is lack of description in caption of Fig 2.
