# Supplementary material for: Quantifying the rise and fall of scientific fields
Source: PLoS One. 2022 Jun 23;17(6):e0270131. doi: 10.1371/journal.pone.0270131 (PMC9223313; doi:10.1371/journal.pone.0270131)
Supplement: Attachment — Submitted filename: Response to Reviewers.pdf [file pone.0270131.s004.pdf]

RESPONSE TO REVIEWERS

We thank the Editor for the opportunity to revise and resubmit the manuscript, and the Reviewers for their constructive comments and suggestions that have helped improve the manuscript. We have incorporated the suggestions, and in particular we significantly expanded the introduction and discussion of the manuscript . We detail our various changes below in a point-by-point answer.

REVIEWER 1

Reviewer #1: Summary

This paper reveals the common pattern in research productivity over the lifecycle of academic fields by analyzing millions of papers submitted to arXiv, the open-access repository of digital preprints, over two decades (Fig. 1). Annual research productivity in a field changes over time, following a growth curve of “fast increase and slow decay (Fig. 2),” during which process small teams dominate the first stage by combining distant, interdisciplinary ideas to create theories, methods, and paradigms, pushing the field towards the peak of productivity, whereas large teams dominate the second stage by citing, developing, and fine-tuning what has been created, leading to the maturity and eventually decline of the field (Fig. 3).

Contributions

The contribution is threefold 1) identifying the common patterns of productivity life cycle across fields, which allows for the effective comparison between fields of different history length and distinct productivity levels in search of universal mechanisms of attention shifting and knowledge production; 2) linking team characteristics and contribution to field development, demonstrating how small and young teams working on broad topics are followed by large and old teams working on specialized topics; 3) quantifying the cognitive distance between fields to support team interdisciplinary analysis, which eases the concerns that some fields are closer than others.

Recommendation

The method is solid, the writing is fluent, and the organization of main figures and supportive materials is clear. Meanwhile, the introduction and discussion sessions can be strengthened. I recommend major revision. My assessments and suggestions for which I expect point-by-point responses are shown below.

We thank the Reviewer for their constructive assessment of our manuscript. We have now expanded the introduction and discussion sections of the manuscript. Moreover, we provide below our answers to the various points raised.

Assessments and suggestions

I used the following checking items to guide my assessment in addition to the journal's rubric.

1. Research question non-trivial?

Yes. The pattern of field development and its association with individual research choices is an important question with both theoretical meanings (organization of scientific knowledge) and policy implications (national funding strategies to develop fields).

2. Assumption rationale insightful?

No. A convincing assumption rationale is not presented. The current rationale reads like "because Gumbel is used a lot elsewhere so we assume it may also work here" (line 73-74). I would encourage unfolded discussions on why "fast increase and slow decay" should be anticipated if Gumbel is pre-determined as the best candidate. Otherwise, Gumbel is discovered rather than expected, then alternative distributions should be compared. The best is to do both.

Response:

We thank the reviewer for this valuable suggestion. We have rephrased the corresponding paragraph in the revised manuscript supporting our use of a right-skewed distribution, in particular citing previous literature on epidemiological models of the evolution of research fields (ref 29). We have also added a comparison with other distributions (Supplementary Fig SI 4, see also below). We observed that the two-parameter Gumbel distribution performs marginally better than other distributions, both using KS test as well as mean squared error. In particular, we show that the Gumbel distribution provides a better account for the fit in the tail of the distribution (Fig SI 4b).

3. Assumption testing solid?

Yes, but could be better. The authors only calculate the goodness of fit for the Gumbel distribution rather than comparing Gumbel against other possible candidates. For example, why is Poisson distribution, which is similarly skewed but has fewer parameters, not considered? Without comparing against other distribution of similar shapes, it is hard to understand what the Gumbel model uniquely contributes. While the distribution fitting and comparison can be improved, the comparison against random-shuffle models in presenting the association between team characteristics and field stage is well done (Figure 3).

Response:
We thank the reviewer for highlighting these points. As said in the previous comment, we added comparisons with the Poisson, lognormal, Weibull, as well as Gaussian distributions. We also elaborated on previously proposed epidemiological models (ref 29)

that produce such extreme value distributions to better support the reasoning underlying our assumption.

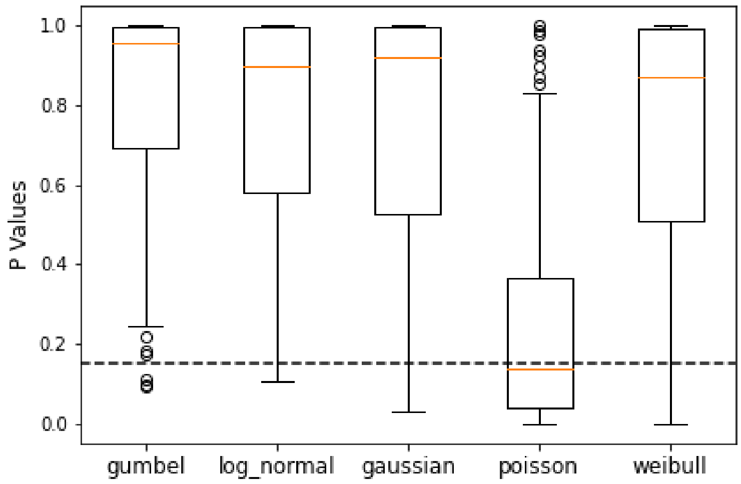

a)

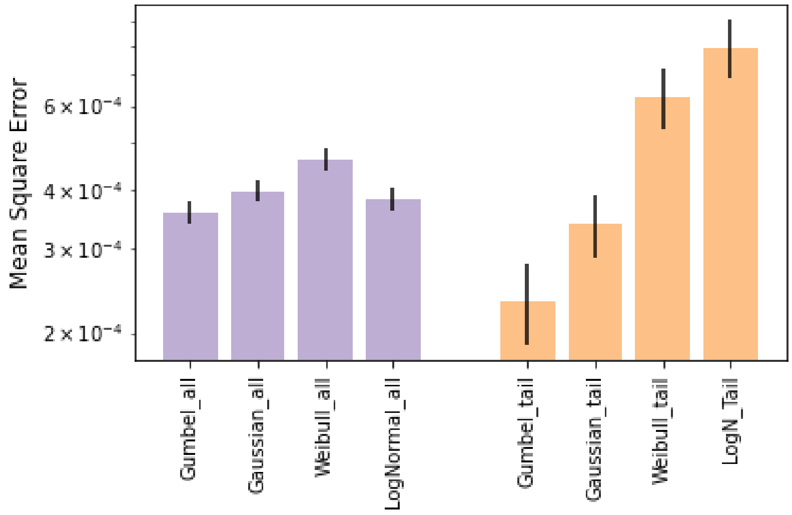

b)

Fig. S4 : (a) We show the p-values for the KS-test statistic considering different distributions for fitting the temporal distributions for all fields studied. Here higher p-values mean a better fit. We observe that the two-parameter functions perform better than single parameter functions, with Gumbel providing generally improved fits over Gaussian, LogNormal and Weibull. (b) We show in addition the mean squared error for all data points (purple) and the tail of the distributions, defined as the 5% of points in the highest rescaled time range (orange). We observe that the Gumbel distribution outperforms other distributions in the tails.

4. Literature review relevant and extensive?

Yes, but weak. The author claimed *"we are still lacking a unified framework to delineate stereotyped stages in the evolution of scientific fields that can be validated over a large number of well-annotated research fields. (Line 33)"* This is not true. While I agree that large-scale empirical tests are rare, theoretical framework on the development of scientific fields is not rare. Randall Collins's theory on "high-consensus, rapid-discovery science" (1994, https://link.springer.com/article/10.1007/BF01476360) is the most relevant to this study. Also the work by Moody & White (2003, https://www.jstor.org/stable/3088904) in analyzing field cohesion. Recent work measuring the "paradigmaticness" of disciplines by Evans et al. (2016, https://sociologicalscience.com/articles-v3-32-757/) from an NLP perspective could also be a good complement to the network perspective of this work.

Response:

We thank the reviewer for these useful references. They add value to our work and have been incorporated in the revised manuscript.

5. Large and novel datasets that may contribute to the community?

Yes, but could be better. The author used arXiv which is open-access datasets and specified the API in the Methods section (URL should be provided). The best is to also share processed datasets to maximize the impact of this work through supporting future explorations.

Response:

We thank the reviewer for the suggestion. We have added the link to the open-access API and a link to the processed dataset in the revised manuscript. We have also added links to some alternative sources for the arXiv dataset and other useful github repositories.

Additional suggestions:

6. If the authors want to maximize audience appreciation, maybe want to consider getting rid of "scale invariant?" This term comes from physics where pattern universality across scales is appreciated as a default. For scholars from bibliometrics, information science, policy scholars, and more, who do not necessarily have this background but could potentially be interested in this paper, this word may push them away. The use of this word implies that (field) size is the main "trouble" in understanding knowledge organization so that if the size is controlled, the major challenge is solved. But large and small fields may have distinct dynamics that not necessary can or should be cheaply "controlled". See recent discussion on slowed canonical progress in large fields by Chu & Evans (2018, https://www.pnas.org/content/118/41/e2021636118). I would encourage considering sticking to "common patterns," but this is a rather personal (writing-style) suggestion that authors should feel free to ignore.

Response: We thank the reviewer for their suggestion to ease the readability of the manuscript for a wider audience. Accordingly, we have incorporated the word "common patterns" in the revised manuscript.

7. Somewhere in the paper, the Diffusion of Innovation Theory by Rogers (1962) (https://en.wikipedia.org/wiki/Diffusion_of_innovations) should be cited. Rogers is well-acknowledged by proposing the "s-curve" theory of diffusion and firstly identified innovators, early adopters, early majority, late majority, and laggards, which seem very similar to the five stages proposed by the authors.

Response:

We agree with the reviewer's suggestion. We did cite Rogers' book in our original manuscript [24]. However the citation needed to be corrected, which we have done in the revised manuscript. We thank the reviewer for highlighting this point.

8. The shift peak of arXiv research interests from physics to computer science and economy is worth saying more (line 64). Is it because of the increase in research demand (problems, grants, and publication opportunities) or caused by the increase in research supply (e.g., talent spillover from physics, which attracted and trained capable researchers but does not necessarily offer enough relevant jobs)?

Response:

We thank the reviewer for highlighting this point. Indeed arXiv submissions have become more diverse with increasing submissions from Computer Science and Economics, indicating a shift away from its initial focus on Physics fields. Yet we lack more comprehensive data to reveal the underlying causes of this shift. Following the Reviewer's comment, we added this point as a target for future research and suggested several potential drivers in the discussion section.

9. The meaning of parameters alpha and beta should be discussed in the field development context (line 78).

Response:

In Equation 1 of the original manuscript alpha and beta parameters are the location and scale parameters respectively that control the shape of the Gumbel distribution. To get more insights on the role of the alpha and beta parameters, we show in Fig SI 3 some examples of Gumbel distributions with varying parameters. The parameter alpha corresponds to the peak location, while the parameter beta corresponds to the width and scale of the distribution. Fields with a low beta have a rapid rise followed by a rapid decay with a long tail. These typically can be the fields promoted by sudden advances in science and technologies or economics, for example, the field "Pricing of Securities in Quantitative finance" (q-fin.pr) (Fig SI 3c). On the other hand fields with a large beta have a longer gradual rise and fall with a long tail in the decay phase, for example, the field "Condensed

Matter Material Sciences” (cond-mat.mtrl-sci) (Fig SI 3d). We have now added this information in the main text.

10. The method authors used to model cognitive distance between fields is a network model (within which what the edge weight represented should be explained) but not “network embedding (line 153),” which is to learn the vector presentation of nodes and edges by extending word2vec (Mikolov, 2013). This differentiation is important because there is emerging work on quantifying cognitive distance between fields using network embeddings, such as (Peng et al. 2021: https://www.science.org/doi/10.1126/sciadv.abb9004) and (Lin et al., https://arxiv.org/abs/2103.03398).

Response:
We thank the reviewer for highlighting this point and pointing to these interesting references. We agree with the reviewer that using the phrasing “network construction” is better suited than “network embedding”. We have corrected this in the revised manuscript. We have also added an expanded explanation on our method of defining the network weights and cognitive distance in the Methods section of the revised manuscript. More sophisticated network embeddings could be used in further research.

11. The thresholds used to identify five stages seems arbitrary: “2.5%, 16%, 50% and 84% (line 104).” How these numbers are picked against “natural options” like 20%, 40%, 60%…should be justified. These numbers are very similar to the five numbers used by Rogers (https://en.wikipedia.org/wiki/Diffusion_of_innovations). If so, Rogers’ book should be cited with numbers explained.

Response: Indeed we borrow these thresholds from Rogers “Diffusion of Innovation theory” (line 101 in the original manuscript). We have corrected the citation [24] in the old manuscript to Rogers book “Diffusion of Innovations” citation [30] and discussed the thresholds in the revised manuscript.

12. How the author’s career stage is associated with the field stage should be explained better. For papers, it is straightforward to understand which field stage it belongs to because a paper has a publication year. But many scholars have a career length equal to or even longer than the analyzed time period (two decades).

Response: We thank the reviewer for the comment. We would like to highlight that here the author's career stage is within arXiv. For every author we arrange their papers in arXiv chronologically and define the career stage as the relative rank of the manuscript (Equation 3 of the revised manuscript). This way for every paper we can map the current stage of its contributing authors careers given their total publications in arXiv. Since each paper can also be categorized into a field stage, we can then relate the authors' career stages to field development stages.

REVIEWER 2

This paper tackles the question on quantifying the dynamics of various scientific fields. The question is addressed by performing a statistical analysis and modeling of a dataset of well-annotated fields tags in Computer science, Mathematics, Finance and Biology, where for 1.45 million articles from 175 research fields in arXiv repository are used. The authors use Gumbel distribution as a proxy to model the field temporal distributions. It is a new and simple scale-invariant methods in quantifying evolution of scientific fields. Then the statistical analysis shows the general characteristics of articles and authors across evolution stages of fields: the early phase is represented by the mixing of cognitively distant fields by small teams of interdisciplinary authors, while late phases exhibit the role of specialized, large teams building on the previous works in the field.

The method and results are presented well using appropriate metrics and figures. The claims of the paper are supported by the results.

Everything looks technically correct and the language is clear.

Response:

We thank the reviewer for their comments and provide our comments below.

I have two major issues and a few minor concerns regarding the analysis and interpretation of some of the results:

Major issues:

1. In order to control the growth effect over time of arXiv articles, the authors use yearly proportion of articles of considered field in arXiv as a metric to delineate the temporal characters instead of absolute number. My concern on this metric is that papers in some big fields will be like to be overwhelmed to small fields resulting in the misunderstanding of real evolution trend of small fields. For example, we can see from fig 2d, Computer science recently enters in decay stage. However, the empirical intuition is that computer science is booming specially in AI field. The phenomenon could also happen in other fields. The authors are suggested to further clarify the reasonable usage of this metric to eliminate reader's worries.

Response:

We thank the reviewer for their comment. Our motivation behind using yearly proportions of articles was to measure the relative growths of fields in this scientific ecosystem where the total number of fields is fixed (as seen in (b) below). In such a closed system, the share of papers indicates the importance of a field within the arXiv community. While the example of Fig 2d does show a decay for one field in CS, we note that most tags dedicated to CS (including AI) are indeed booming in our analysis, as can be seen from

the heatmap in Fig 1b (see zoomed version in (a) below). This figure has also been added to the supplementary information.

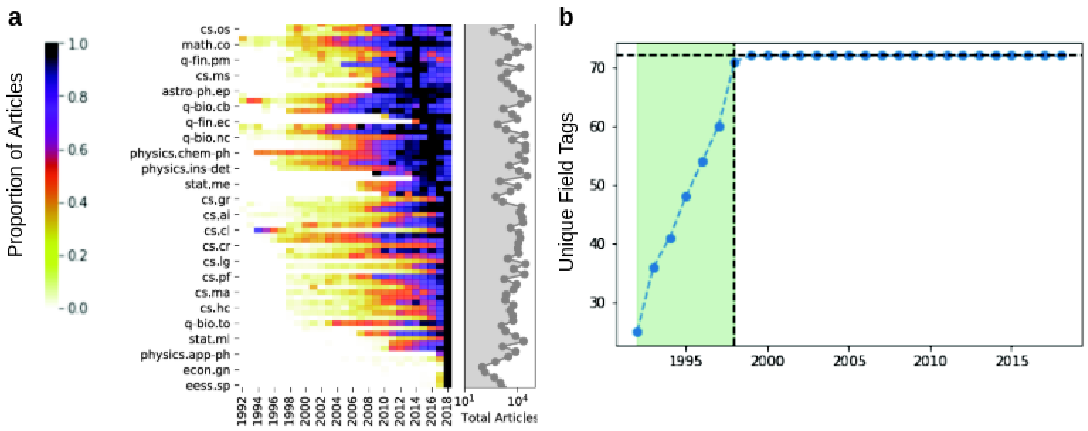

Caption: (a) Zoom of histogram in Fig. 1b of the main manuscript exhibits peaking of field tags from the field of computer science in later years, including CS.AI (artificial intelligence). (b) Cumulative number of field tags across time, among the 72 studied tags. After an initial growth in the early years, the number of unique tags stays constant.

2. By statistical analysis highlighting characteristics shared by articles and authors across various stages of a field evolution, the authors draw one of the main conclusions: early stages are characterized by small teams and interdisciplinary authors, while late stages exhibit the role of large, more specialized teams. They wrote in Discussion section of the paper “This supports the general finding that small teams disrupt while large teams develop science and technology [33, 34].” Although I agree the narration above, I also suggest that the authors make an extra effort to calculate the Disruptiveness Index to support their findings.And there is a recently published paper closely related to the topic in this paper, it is recommended to be added in the reference list.

Johan S. G. Chu, James A. Evans. (2021). Slowed canonical progress in large fields of science. Proc Natl Acad Sci, 41(118), e2021636118.

Response:

We thank the reviewer for their suggestion, which provided an interesting novel result to our manuscript. We calculated the Disruptive Index considering the citation network across arXiv papers (see Methods section). The figure below shows the Disruptive Index computed across all articles in different stages of evolution compared against random shuffling of timestamps. As was expected from the discussion, we observe that the earlier stages have more disruptive articles than later stages, with a steady decrease over field development. We now added this result as a new Fig 4.

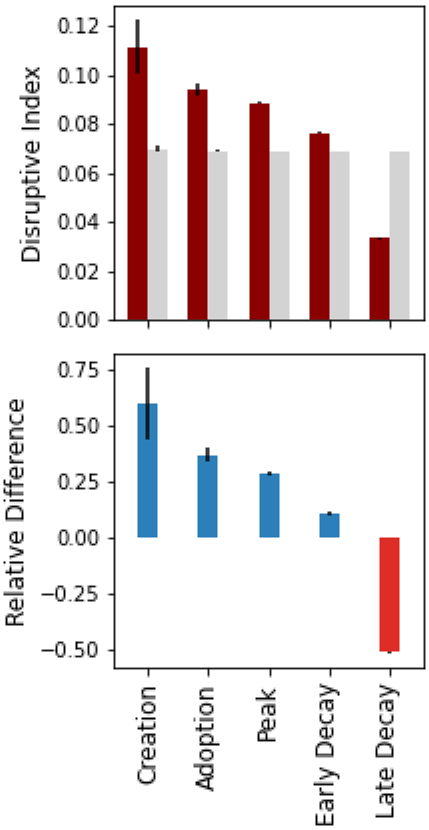

Fig 4. Disruptive index measure for the articles in arXiv. The observed values are averaged over all articles (red bars). Gray bars correspond to the average random expectation (see Methods). The bottom plot represents the relative difference between observed and random values. Error bars denote standard errors. Earlier stages have more disruptive articles, followed by a steady decrease in later stages.

Our observation is in line with the general finding in reference [39] of the revised manuscript. However, we note that there are two limitations in this computation: (1) the citation network in our dataset only includes citations within arXiv, which are sparser compared to more exhaustive datasets, and (2) the behavior of citing arXiv articles over published manuscripts might be field dependent. These limitations are now discussed in the revised manuscript.

We have included the above figure in the revised manuscript. We have also included the recommended reference in the revised manuscript.

A few minor concerns:

3.The paper uses re-scaled Gumbel distribution to model the rise and fall of a scientific filed. I have a detailed doubt about the calculation of stages. In fig 2b and fig 2d, if they use the 25%, 16%, 50% and 84% quantiles of the Gumbel distribution as cutpoints of

stage, the math is recently on the decay stage. But at 84% quantiles, it is around the peak rather than late decline in fig 2b. Maybe I miss the details about the method. The authors are suggested to explain this point clearly.

Response:

We thank the Reviewer for their comment to help clarify our message. In order to rescale the fields, we first fit individual fields to the Gumbel distribution. We then use the location and scale parameters from the fitted function to rescale the timeline for each field using Eq. 2 in the revised manuscript, resulting in the aggregated distributions as shown in Fig. 2e of the revised manuscript. The quantiles for cut-off are obtained from the fitted blue curve in Fig. 2e, i.e. incorporating information from all articles. These cut-offs define the stage buckets that are used for all fields. We then categorize article centric and author centric properties for each field and represent the average properties and comparison against the random across fields in Figure 3.

We have rephrased the subsection “Characterizing the stages of research field evolution” to elaborate more clearly on the calculation of stages in the manuscript and on the method we used.

4.Under strict conditions for data: longevity, unimodality and completeness, 175 research fields are reduced the number of fields to 72. The exiting rate of fields is bigger than ½, which means the authors lose and neglect lots of information in the main text. In my opinion, while it is much meaningful to find the scale-invariant patterns in the evolution of many scientific fields, the manuscript can be better if the authors clearly highlight the limitation in Discussion. The evolution characters of 72 fields in the paper cannot stand for the pattern of all fields in science. And it is worth exploring the untrivial patterns in further research.

Response: We thank the reviewer for the suggestion. This has been added as a limitation of the current study in the discussion section of the revised manuscript.

5. In fig 1b, the authors compare the publishing seasonality between weekdays and weekends while the information is not mentioned or discussed at all to support the results in the manuscript. In my opinion, it is redundant and useless for publication.

Response: We thank the reviewer for highlighting this. We have moved figure 1b to the Supplementary information.

6. The Fig 2d is lack of description in caption of Fig 2.

Response: This has been corrected in the revised manuscript.

Funding Statement

The author(s) received no specific funding for this work. Thanks to the Bettencourt Schueller Foundation long term partnership, this work was partly supported by the CRI Research Fellowship to MS. Nokia bell labs provided support in the form of salaries for author LT, but did not have any additional role in the study design, data collection and analysis, decision to publish, or preparation of the manuscript. The specific roles of these authors are articulated in the 'author contributions' section.
