# Supplementary material for: Quantifying the rise and fall of scientific fields
Source: PLoS One. 2022 Jun 23;17(6):e0270131. doi: 10.1371/journal.pone.0270131 (PMC9223313; doi:10.1371/journal.pone.0270131)
Supplement: Attachment — Submitted filename: Quantifying scientific fields_PONE-D-21-36265_Lingfei WuReview.docx [file pone.0270131.s002.docx]

**Summary**

This paper reveals the common pattern in research productivity over the lifecycle of academic fields by analyzing millions of papers submitted to arXiv, the open-access repository of digital preprints, over two decades (Fig. 1). Annual research productivity in a field changes over time, following a growth curve of “fast increase and slow decay (Fig. 2),” during which process small teams dominate the first stage by combining distant, interdisciplinary ideas to create theories, methods, and paradigms, pushing the field towards the peak of productivity, whereas large teams dominate the second stage by citing, developing, and fine-tuning what has been created, leading to the maturity and eventually decline of the field (Fig. 3).

**Contributions**

The contribution is threefold 1) identifying the common patterns of productivity lifecycle across fields, which allows for the effective comparison between fields of different history length and distinct productivity levels in search of universal mechanisms of attention shifting and knowledge production; 2) linking team characteristics and contribution to field development, demonstrating how small and young teams working on broad topics are followed by large and old teams working on specialized topics; 3) quantifying the cognitive distance between fields to support team interdisciplinary analysis, which eases the concerns that some fields are closer than others.

**Recommendation**

The method is solid, the writing is fluent, and the organization of main figures and supportive materials is clear. Meanwhile, the introduction and discussion sessions can be strengthened. I recommend *major revision*. My assessments and suggestions for which I expect point-by-point responses are shown below.

**Assessments and suggestions**

I used the following checking items to guide my assessment in addition to the journal’s rubric.

1. Research question non-trivial?

Yes. The pattern of field development and its association with individual research choices is an important question with both theoretical meanings (organization of scientific knowledge) and policy implications (national funding strategies to develop fields).

2. Assumption rationale insightful?

No. A convincing assumption rationale is not presented. The current rationale reads like “because Gumbel is used a lot elsewhere so we assume it may also work here” (line 73-74). I would encourage unfolded discussions on why “fast increase and slow decay” should be anticipated if Gumbel is pre-determined as the best candidate. Otherwise, Gumbel is discovered rather than expected, then alternative distributions should be compared. The best is to do both.

3. Assumption testing solid?

Yes, but could be better. The authors only calculate the goodness of fit for the Gumbel distribution rather than comparing Gumbel against other possible candidates. For example, why is Poisson distribution, which is similarly skewed but has fewer parameters, not considered? Without comparing against other distribution of similar shapes, it is hard to understand what the Gumbel model uniquely contributes. While the distribution fitting and comparison can be improved, the comparison against random-shuffle models in presenting the association between team characteristics and field stage is well done (Figure 3).

4. Literature review relevant and extensive?

Yes, but weak. The author claimed “we are still lacking a unified framework to delineate stereotyped stages in the evolution of scientific fields that can be validated over a large number of well-annotated research fields. (Line 33)” This is not true. While I agree that large-scale empirical tests are rare, theoretical framework on the development of scientific fields is not rare. Randall Collins’s theory on “high-consensus, rapid-discovery science” (1994,<https://link.springer.com/article/10.1007/BF01476360> ) is the most relevant to this study. Also the work by Moody & White (2003,<https://www.jstor.org/stable/3088904> ) in analyzing field cohesion. Recent work measuring the “paradigmaticness” of disciplines by Evans et al. (2016,<https://sociologicalscience.com/articles-v3-32-757/> ) from an NLP perspective could also be a good complement to the network perspective of this work.

5. Large and novel datasets that may contribute to the community?

Yes, but could be better. The author used arXiv which is open-access datasets and specified the API in the Methods section (URL should be provided). The best is to also share processed datasets to maximize the impact of this work through supporting future explorations.

Additional suggestions:

6. If the authors want to maximize audience appreciation, maybe want to consider getting rid of “scale invariant?” This term comes from physics where pattern universality across scales is appreciated as a default. For scholars from bibliometrics, information science, policy scholars, and more, who do not necessarily have this background but could potentially be interested in this paper, this word may push them away. The use of this word implies that (field) size is the main “trouble” in understanding knowledge organization so that if the size is controlled, the major challenge is solved. But large and small fields may have distinct dynamics that not necessary can or should be cheaply “controlled”. See recent discussion on slowed canonical progress in large fields by Chu & Evans (2018,<https://www.pnas.org/content/118/41/e2021636118> ). I would encourage considering sticking to “common patterns,” but this is a rather personal (writing-style) suggestion that authors should feel free to ignore.

7. Somewhere in the paper, the Diffusion of Innovation Theory by Rogers (1962) (<https://en.wikipedia.org/wiki/Diffusion_of_innovations>) should be cited. Rogers is well-acknowledged by proposing the “s-curve” theory of diffusion and firstly identified innovators, early adopters, early majority, late majority, and laggards, which seem very similar to the five stages proposed by the authors.

8. The shift peak of arXiv research interests from physics to computer science and economy is worth saying more (line 64). Is it because of the increase in research demand (problems, grants, and publication opportunities) or caused by the increase in research supply (e.g., talent spillover from physics, which attracted and trained capable researchers but does not necessarily offer enough relevant jobs)?

9. The meaning of parameters alpha and beta should be discussed in the field development context (line 78).

10. The method authors used to model cognitive distance between fields is a network model (within which what the edge weight represented should be explained) but not “network embedding (line 153),” which is to learn the vector presentation of nodes and edges by extending word2vec (Mikolov, 2013). This differentiation is important because there is emerging work on quantifying cognitive distance between fields using network embeddings, such as (Peng et al. 2021:<https://www.science.org/doi/10.1126/sciadv.abb9004>) and (Lin et al.,<https://arxiv.org/abs/2103.03398>).

11. The thresholds used to identify five stages seems arbitrary: “2.5%, 16%, 50% and 84% (line 104).” How these numbers are picked against “natural options” like 20%, 40%, 60%…should be justified. These numbers are very similar to the five numbers used by Rogers (<https://en.wikipedia.org/wiki/Diffusion_of_innovations>). If so, Rogers’ book should be cited with numbers explained.

12. How the author’s career stage is associated with the field stage should be explained better. For papers, it is straightforward to understand which field stage it belongs to because a paper has a publication year. But many scholars have a career length equal to or even longer than the analyzed time period (two decades).
